# MiR-148a, a microRNA upregulated in the WNT subgroup tumors, inhibits invasion and tumorigenic potential of medulloblastoma cells by targeting Neuropilin 1

**DOI:** 10.18632/oncoscience.137

**Published:** 2015-03-02

**Authors:** Kedar Yogi, Epari Sridhar, Naina Goel, Rakesh Jalali, Atul Goel, Aliasgar Moiyadi, Rahul Thorat, Pooja Panwalkar, Atul Khire, Archya Dasgupta, Prakash Shetty, Neelam Vishwanath Shirsat

**Affiliations:** ^1^ Shirsat Laboratory, Advanced Centre for Treatment, Research and Education in Cancer, Tata Memorial Centre, Kharghar, Navi Mumbai, India; ^2^ Department of Pathology, Advanced Centre for Treatment, Research and Education in Cancer, Tata Memorial Centre, Kharghar, Navi Mumbai, India; ^3^ Department of Surgical Oncology, Advanced Centre for Treatment, Research and Education in Cancer, Tata Memorial Centre, Kharghar, Navi Mumbai, India; ^4^ Department of Radiation Oncology, Tata Memorial Hospital, Tata Memorial Centre, Parel, Mumbai, India; ^5^ Laboratory Animal Facility, Advanced Centre for Treatment, Research and Education in Cancer, Tata Memorial Centre, Kharghar, Navi Mumbai, India; ^6^ Department of Pathology, Seth G. S. Medical College and K. E. M. Hospital, Parel, Mumbai, India; ^7^ Department of Neurosurgery, Seth G. S. Medical College and K. E. M. Hospital, Parel, Mumbai, India

**Keywords:** Medulloblastoma, miR-148a, Neuropilin 1, invasion, WNT subgroup

## Abstract

Medulloblastoma, a common pediatric malignant brain tumor consists of four molecular subgroups viz. WNT, SHH, Group 3 and Group 4. MiR-148a is over-expressed in the WNT subgroup tumors, which have the lowest incidence of metastasis and excellent survival among all medulloblastomas. MiR-148a was expressed either in a transient manner using a synthetic mimic or in a stable doxycycline inducible manner using a lentiviral vector in non-WNT medulloblastoma cell lines. Expression of miR-148a to levels comparable to that in the WNT subgroup tumors was found to inhibit proliferation, clonogenic potential, invasion potential and tumorigenicity of medulloblastoma cells. MiR-148a expression in medulloblastoma cells brought about reduction in the expression of NRP1, a novel miR-148a target. Restoration of NRP1 expression in medulloblastoma cells was found to rescue the reduction in the invasion potential and tumorigenicity brought about by miR-148a expression. NRP1 is known to play role in multiple signaling pathways that promote tumor growth, invasion and metastasis. NRP1 expression in medulloblastomas was found to be associated with poor survival, with little or no expression in majority of the WNT tumors. The tumor suppressive effect of miR-148a expression accompanied by the down-regulation of NRP1 makes miR-148a an attractive therapeutic agent for the treatment of medulloblastomas.

## INTRODUCTION

Brain tumors are the most common solid tumors in children. Medulloblastomas account for about 20% of all primary brain tumors in children. Standard treatment includes surgical resection, followed by cranio-spinal radiation and chemotherapy. Advances in surgical and radiation techniques have improved the 5-year survival rate to about 80% for average risk patients and 55-76% for high risk patients [1]. Genome wide expression profiling studies have identified four core molecular subgroups of medulloblastomas called WNT, SHH, Group 3 and Group 4 [2]. WNT and SHH subgroup tumors appear to result from the constitutive activation of WNT and SHH signaling pathways respectively, based on their expression profiles as well as their mutational landscape [2, 3]. Group 3 and Group 4 tumors have overlapping gene expression profiles. MYC over-expression/amplification and expression of the photoreceptor signaling pathway genes is a characteristic of most Group 3 tumors while Group 4 tumors are characterized by the expression of various neuronal differentiation related genes [2, 4]. The four molecular subgroups also vary in clinical characteristics like age related incidence, presence of metastasis and survival. Group 3 tumors have the highest incidence of metastasis at diagnosis and poor overall survival. WNT subgroup medulloblastomas on the other hand have the lowest incidence of metastasis at diagnosis (< 10%) and highest survival (90% - 95% ten year overall survival) among all the four subgroups [5].

We have earlier reported that WNT subgroup tumors have a distinctive microRNA profile with a number of miRNAs over-expressed in these tumors as compared to other medulloblastoma subgroups as well as normal cerebellar tissues [4]. Further, over-expression of miR-193a-3p, miR-224, miR-148a, miR-23b, and miR-365 in the WNT subgroup tumors was validated in a study done on 103 medulloblastomas [6]. While miR-148a is upregulated in the WNT subgroup medulloblastomas, it is known to be downregulated as a result of promoter hypermethylation in various cancers and has been shown to act as tumor-suppressive/metastasis suppressive miRNA [7, 8]. It is therefore likely that miR-148a contributes to lower metastatic incidence and excellent survival of the WNT subgroup medulloblastomas. In order to understand role of miR-148a in medulloblastoma biology, effect of miR-148a expression on the growth and malignant behavior of established medulloblastoma cell lines was investigated in the present study.

## RESULTS

**Figure 1:**
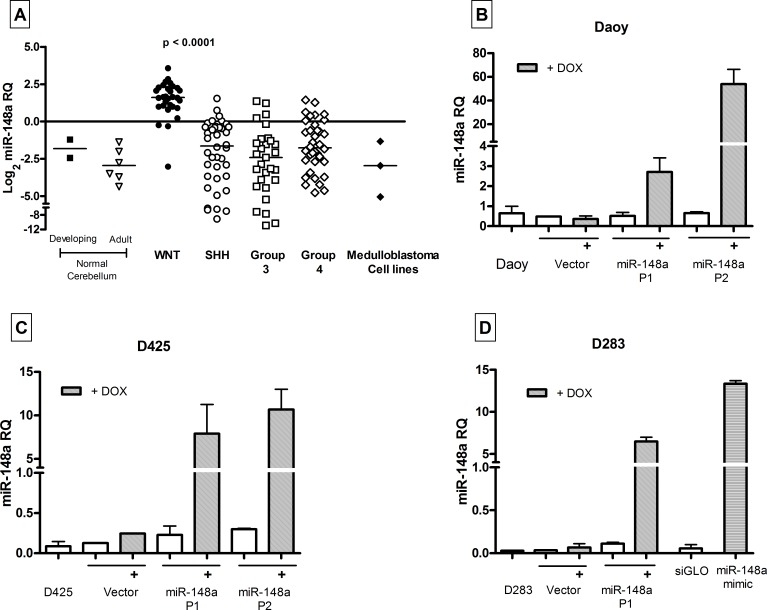
Expression levels of miR-148a across four molecular subgroups of medulloblastoma, cell lines and miR-148a expressing stable polyclonal populations, as determined by real time RT-PCR assay (A) miR-148a levels were evaluated in 141 medulloblastoma tissues classified in the four molecular subgroups, normal developing (from less than 1year old infants) cerebellar tissues and adult (from individuals >21 yr of age) cerebellar tissues. (B, C, D) Relative Quantity of miR-148a in the indicated medulloblastoma cell line and its polyclonal populations transduced with pTRIPZ control lentiviral vector (Vector) or pTRIPZ-miR-148a construct expressing miR-148a in doxycycline inducible manner (P1, P2) and in the cells transiently transfected with control siGLO or miR-148a mimic. + DOX: doxycycline induced.

MiR-148a expression was studied in a total of 141 medulloblastoma tumor tissues (including 103 cases reported earlier) by real time RT-PCR analysis. The tumor tissues were molecularly classified using a panel of 12 protein-coding genes and 9 miRNAs for molecular classification as described before [6]. WNT subgroup medulloblastomas were found to over express miR-148a by 6-12 fold (p < 0.0001) as compared to the other subgroup tumors, 10-20 fold as compared to normal cerebellar tissues and 7-100 fold as compared to the established medulloblastoma cell lines (Figure 1A). In order to understand the role of miR-148a in medulloblastoma biology, medulloblastoma cell lines were transduced with pTRIPZ lentiviral vector expressing miR-148a in doxycycline inducible manner. D425 and D283 cell lines belong to Group 3, Group 3/Group 4 medulloblastoma subgroup respectively while Daoy cell line belongs to SHH subgroup based on their cytogenetic profiles [9, 10] and expression profiles (data not shown).

### Effect of miR-148a expression on the growth of medulloblastoma cells studied using MTT assay

**Figure 2:**
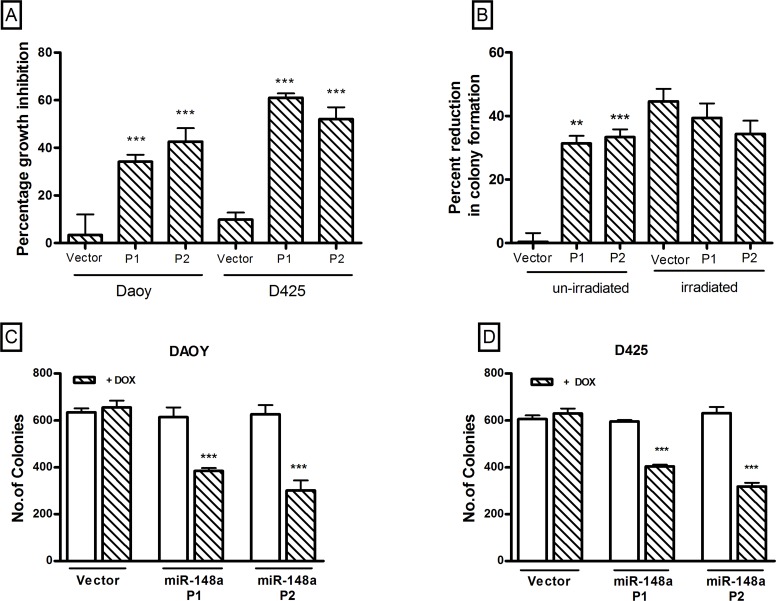
Effect of miR-148a expression on growth, clonogenic potential and, anchorage independent growth of medulloblastoma cell lines studied using stable polyclonal populations (P1, P2) of Daoy and D425 cells expressing miR-148a on doxycycline induction (A) Y axis denotes percentage growth inhibition obtained on doxycycline induction of the vector control and P1, P2 polyclonal populations of the indicated cell line as judged by the MTT assay. (B) Y axis indicates percent reduction in the number of colonies formed on doxycycline induction of the vector control and P1, P2 population cells of Daoy cells and percent reduction in the number of colonies formed on irradiation of doxycycline induced indicated population, in a clonogenic assay. (C, D) Y axis denotes the number of colonies formed in a soft agar assay by the vector control and P1, P2 population of the indicated cell line. The significance of the difference in the growth inhibition or soft agar colony formation observed on doxycycline induction of the polyclonal populations expressing pTRIPZ-miR-148a as compared to that observed in the polyclonal population transfected with control pTRIPZ vector was calculated by the student's t test. ** indicates p < 0.001 while *** indicates p < 0.0001 as determined using Student's t test.

Stable polyclonal populations (P1, P2) of medulloblastoma cell lines Daoy and D425 expressing miR-148a in doxycycline inducible manner were selected in the presence of puromycin. Doxycycline treatment of polyclonal population P1 of Daoy cells resulted in the induction of miR-148a expression to a level (RQ: 2.0-4.0) comparable to that in the WNT subgroup tumor tissues while doxycycline induction of P2 polyclonal populations resulted in higher miR-148a expression in the range of RQ: 45-55 (Figure 1B). The effect of miR-148a expression on the growth of the medulloblastoma cell lines was studied over a period of 12 days by MTT assay. Doxycycline induced miR-148a expression resulted in 30-35% and 42-45% growth inhibition of P1 and P2 population respectively (Figure 2A, Supplementary Fig. S1A). Doxycycline induction of P1 and P2 polyclonal populations of D425 cells resulted in the miR-148a expression levels in the range of 5-10 RQ (Figure 1C). MiR-148a expression was found to result in 57-64% growth inhibition of D425 cells (Figure 2A, Supplementary Figure S1B). Thus, miR-148a expression significantly (p < .0001) inhibited growth of both the medulloblastoma cell lines studied.

### Effect of miR-148a expression on clonogenic potential, radiation sensitivity, and anchorage-independent growth of medulloblastoma cell lines

The effect of miR-148a expression on the clonogenic potential and radiation sensitivity was studied using clonogenic assay. MiR-148a expression in Daoy cells was found to reduce clonogenic potential of Daoy cells by 35-40% as studied using both the P1 and P2 polyclonal populations (Figure 2B). Irradiation of vector control cells at a dose of 4 Gy reduced their clonogenic potential by

40-50% with or without doxycycline treatment. Irradiation of Doxycycline induced P1 and P2 population was found to reduce clonogenic potential further by 33-47% (Figure 2B). Thus, miR-148a expression although did not increase radiation sensitivity per se, the combined effect of miR-148a and radiation brought about 80-90% reduction in the clonogenic potential of Daoy cells.

The effect of miR-148a expression on the anchorage-independent growth of medulloblastoma cells was studied by soft agar colony formation assay. The number of soft agar colonies formed by P1 and P2 Daoy cell populations was found to be reduced by 30-45% upon doxycycline induction of miR-148a expression (Figure 2C). The reduction in the soft agar colony formation is comparable to the reduction in the clonogenic potential upon miR-148a expression in Daoy cells. Therefore, miR-148a expression was not found to have significant effect on the anchorage-independent growth potential. Clonogenic potential of D425 cells was studied by soft agar assay as they grow in a semi-suspension manner. Polyclonal populations P1 and P2 of D425 cells showed reduction in the soft agar colony formation by 30-50% upon miR-148a expression (Figure 2D). Thus, miR-148a expression was found to bring about significant (p < 0.0001) reduction in the clonogenic potential of both the medulloblastoma cell lines studied.

### Effect of miR-148a expression on *in vitro* invasion potential of medulloblastoma cell lines

**Figure 3:**
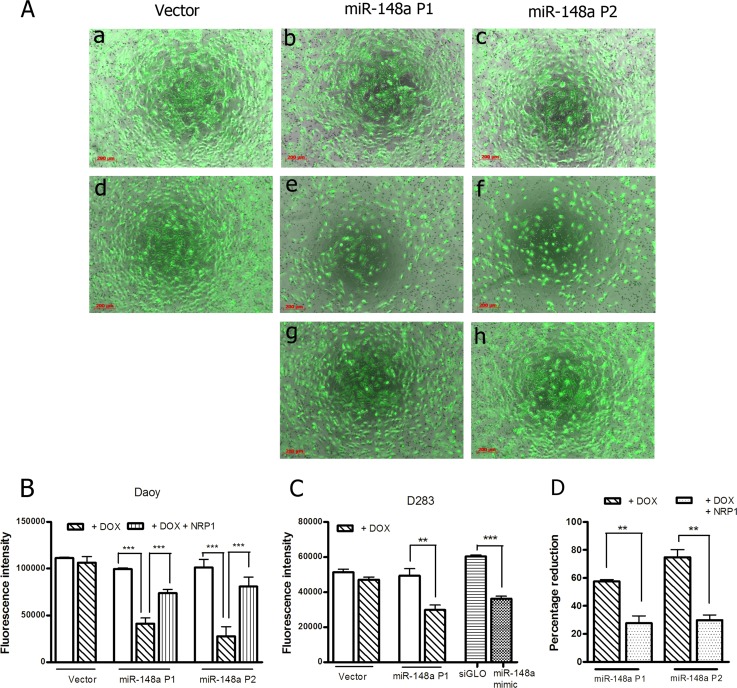
Effect of miR-148a expression on the invasive potential of Daoy and D283 cell lines Invasion potential was determined by studying migration through matrigel coated 8-μm pore size transwell inserts. The study was done using the stable polyclonal populations (P1, P2) of Daoy and D283 cells expressing miR-148a on doxycycline induction. (A) Representative images of the Calcein, AM labeled cells on the lower side of the transwell chamber membrane 36 h after seeding the indicated polyclonal populations of Daoy cells onto the transwell insert, with (d, e & f) or without (a, b & c) induction of miR-148a expression and after restoration of NRP1 expression (g, h) in doxycycline induced cells. (B-C) Y axis denotes the total fluorescence intensity of the invaded cells normalized to the total intensity of the initial cell number seeded of the indicated cell populations. (D) Y axis indicates the percent reduction in the fluorescence of the invaded cells of the indicated stable polyclonal populations on doxycycline induction (+DOX) of Daoy P1 and P2 population cells with (+NRP1) or without restoration of NRP1 expression. ** indicates p < 0.001; *** indicates p < 0.0001.

Effect of miR-148a expression on the invasion potential of medulloblastoma cells was evaluated by studying invasion of the cells through Matrigel^TM^ coated membranes in transwell inserts. MiR-148a expressing polyclonal populations P1 and P2 of Daoy cell line showed 60-70% reduction in the invasion potential upon doxycycline induced miR-148a expression (Figure 3A, B, D). D425 cells were not found to have significant invasion ability through Matrigel^TM^ as studied over a period of 48 h. Therefore, the effect of miR-148a expression on the invasion potential was studied using D283 cells. Stable polyclonal population P1 of D283 cells expressing pTRIPZ-miR-148a was found to express miR-148a at RQ 6-7 on doxycycline induction while transient transfection with miR-148a mimic in D283 cells resulted in miR-148a expression at RQ 12-13.5 (Figure 1D). MiR-148a expression either in a stable inducible manner or in a transient manner using a synthetic miR-148a mimic, showed a reduction in the invasion potential of D283 cells by about 35-43% (Figure 3C).

### Effect of miR-148a expression on *in vivo* tumorigenicity and *in vivo* invasion potential of medulloblastoma cells

**Figure 4:**
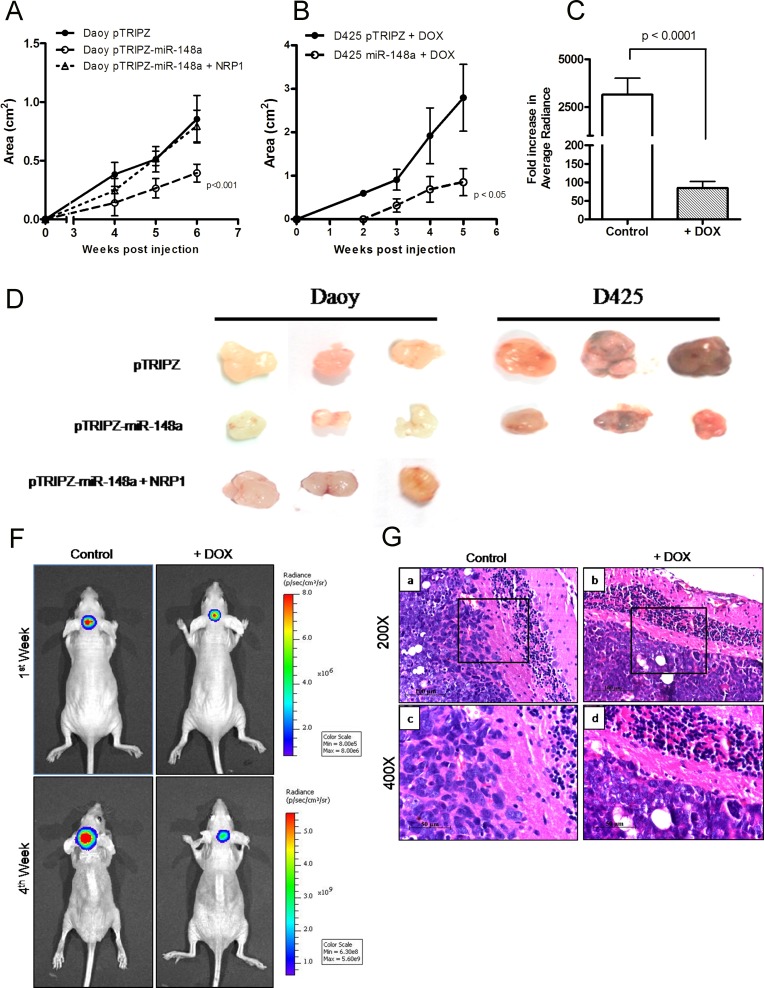
Effect of miR-148a expression on tumorigenicity of medulloblastoma cells (A-B) Y axis denotes the area of subcutaneous tumors formed over a period of 4-6 weeks in nude mice, post injection of doxycycline induced Daoy or D425 P2 polyclonal population (pTRIPZ-miR-148a) and vector (pTRIPZ) control population and after restoration of NRP1 expression in doxycycline induced P2 population of Daoy cells (n = 6 each). (D) shows photographs of these subcutaneous tumors. (F) shows bioluminescence images of nude mice orthotopically injected with D283 stable polyclonal population P1 expressing miR-148a upon doxycycline (DOX) induction and, firefly luciferse under CAG promoter. The images were captured on 1^st^ and 4^th^ week after the injection. (C) Y axis shows relative fold increase in the average radiance on 4^th^ week as compared to that on 1^st^ week after injection of D283 cells P1 population with (+DOX) or without doxycycline induction (Control) in 7 mice each. (G) shows photographs of hematoxylin-eosin stained paraffin sections of the orthotopic xenografts of control and doxycycline induced D283 cells P1 population. The area marked with rectangle in a & b shows invading margins of the tumor cells in the control and doxycycline induced miR-148a expressing cells; c & d show magnified images (400 X) of the invading margins indicated by rectangles in a & b.

In order to study the effect of miR-148a expression on tumorigenicity of medulloblastoma cells, miR-148a expressing polyclonal populations of Daoy and D425 cells were injected subcutaneously in immunodeficient BALB/c Nude mice. Subcutaneous injection of 5 × 10^6^ D425/Daoy cells resulted in tumors of measurable size by 2^nd^and 4^th^ week of injection respectively. MiR-148a expression brought about 45-60% reduction (p < 0.05) in the tumor forming ability of Daoy cell P2 population (Figure 4A, D). Doxycycline induction of miR-148a expression in P2 population of D425 cells was found to bring about 50-80% reduction (p < 0.05) in the size of the tumors formed (Figure 4B, D).

D283 polyclonal population P1 cells, engineered to express firefly luciferase, were injected stereotactically in cerebellum of nude mice with or without doxycycline induction of miR-148a expression. Figure 4C, 4F show~25 fold reduction (p < 0.0001) in the bioluminescence (average radiance) of the tumors formed on induction of miR-148a expression as determined by *in vivo* imaging. Therefore, miR-148a expression was found to decrease tumorigenicity in all the three medulloblastoma cell lines studied. The tumor margin of doxycycline induced (+DOX) miR-148a expressing D283 cells in the cerebellar cortex was cohesive and distinct from the adjacent cerebellar cortex (Figure 4G, B, D). On the other hand, tumor margin of the control D283 cells was much more non-cohesive having loosely spaced cells indicating invasive phenotype (Figure 4G, A, C). Thus, miR-148a expression not only reduced tumorigenicity but also *in vivo* invasion potential of D283 medulloblastoma cells.

### Identification of protein-coding gene targets of miR-148a by Luciferase reporter assay and validation by western blot analysis

**Figure 5:**
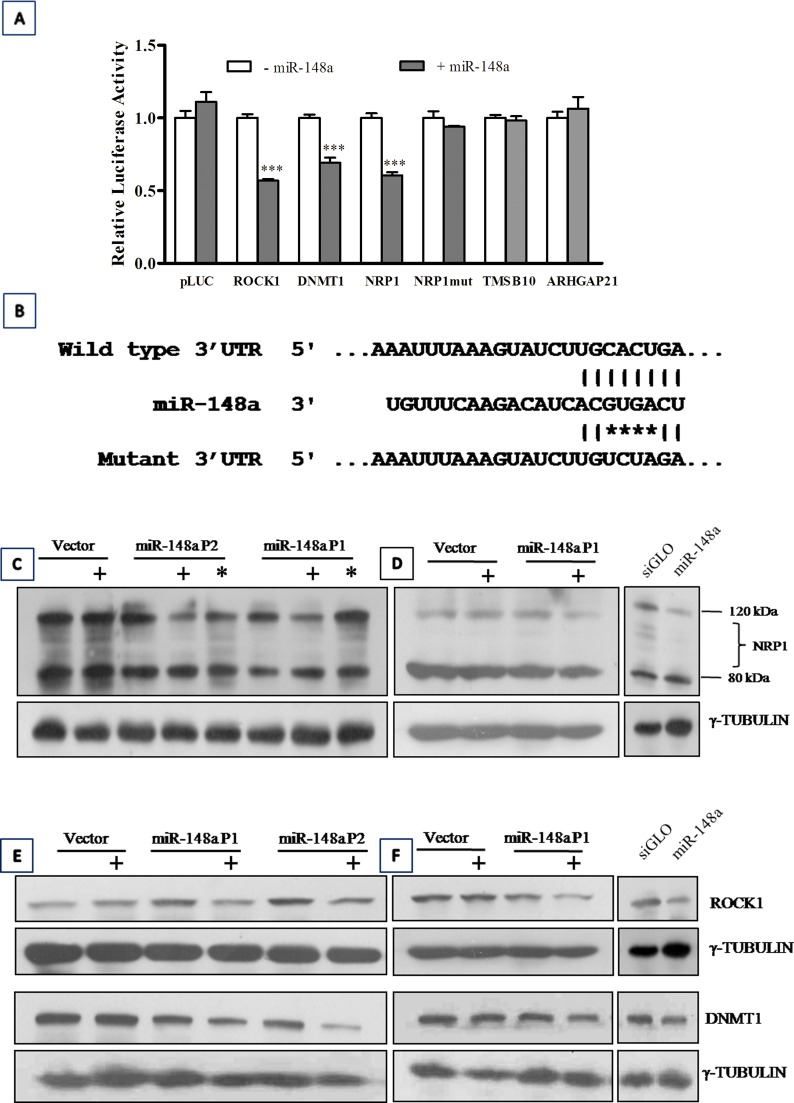
Identification of miR-148a targets Y-axis denotes Luciferase activity of 293 FT cells transfected with the construct having 3′-UTR region of the indicated gene cloned downstream luciferase cDNA in a pcDNA 3.0 vector and EGFP expressing vector with or without pcDNA 3.0 construct expressing miR-148a. The luciferase activity is expressed relative to the GFP fluorescence. *** indicates p < 0.0001. (B) Schematic diagram shows miR-148a target site in NRP1 3′-UTR and position of the mutations (indicated by asterisk) introduced in the target site by site directed mutagenesis. (C, D, E, F) Western blot analysis shows reduction in the expression levels of NRP1, ROCK1 and DNMT1 protein in stable polyclonal populations of Daoy (C, E) and D283 (D, F) cells on doxycycline induction of miR-148a expression. γ-tubulin was used as a loading control. +: Doxycycline induction; * indicates exogenous expression of NRP1 in doxycyline induced cells.

In order to identify miR-148a target genes instrumental in bringing about miR-148a mediated inhibition of invasion and tumorigenic potential, three protein coding genes *viz. NRP1*, *ARHGAP21*, *TMSB10* were investigated as potential miR-148a targets. These genes were predicted as miR-148a targets by the target prediction program, TargetScan 5.01 and were short-listed for experimental validation based on the presence of conserved target site, expression of the target gene in normal cerebellum and medulloblastoma tissues and their known role in invasion/metastasis. 3′-Untranslated regions (3′-UTR) of the putative target genes *viz*. *NRP1*, *ARHGAP21*, *TMSB10* and two known miR-148a target genes *ROCK1* and *DNMT1* were cloned downstream to luciferase cDNA in the reporter vector. Luciferase activity of the cells expressing *ROCK1*, *DNMT1* and *NRP1* constructs was found to be reduced by 30-50% (p < 0. 0001) in the presence of miR-148a expression (Figure 5A) while there was no significant reduction in the luciferase activity of the cells expressing *ARHGAP21*, *TMSB10* constructs. To further confirm *NRP1* as a miR-148a target, miR-148a binding site in the *NRP1* 3′UTR region was mutated by site directed mutagenesis as shown in Figure 5B. The cells expressing *NRP1* 3′UTR mutant construct failed to show reduction in the luciferase activity in the presence of miR-148a.

Western blot analysis showed reduction in the protein levels of NRP1, ROCK1 and DNMT1 in miR-148a expressing P1, P2 populations of Daoy cells and P1 polyclonal population of D283 cells as well as in D283 cells transiently transfected with miR-148a mimic (Figure 5C, D, E, F). The protein levels of the full length 120 kDa NRP1 isoform decreased on miR-148a expression. However, protein levels of the ~ 80 kDa NRP1 isoform were not altered on miR-148a expression. This observation was confirmed in a human glioma cell line U373 that expresses both 120 kDa and 80 kDa NRP1 isoforms at higher levels (Supplementary Figure S2). Since miR-148a targets the 3′-UTR of NRP1 it is likely that the ~80 kDa isoform is not targeted by miR-148a since it lacks the 3′- UTR. Real time RT-PCR analysis was performed using primers for total *NRP1* mRNA expression (all known NRP1 isoforms) as well as primers specific for the 3′- UTR of the full length isoform and primers specific for the transmembrane (TM) region. Relative quantities of NRP1 as estimated using the 3′-UTR specific primers or TM domain specific primers was found to be almost 50% of the total NRP1 expression level in both Daoy and D283 cell line (Supplementary Figure S3A, B). The protein levels of the 120 kDa isoform were also estimated to be about 50% and 30% of the total NRP1 protein levels in Daoy and D283 cell line respectively (Supplementary Figure S3C). Therefore, miR-148a appears to target only the full length NRP1 isoform and not the 80 kDa isoform as it lacks the 3′-UTR.

### Reversal of miR-148a mediated reduction in invasion potential and tumorigenicity of medulloblastoma cells upon exogenous NRP1 expression

MiR-148a expressing polyclonal populations P1 and P2 of Daoy cell line were transfected with a construct expressing NRP1 under Phosphoglycerate kinase 1 (PGK1) promoter (a kind gift from Dr. Lena Claesson-Welsh, Uppsala Universitet, Uppsala, Sweden). The parental P1, P2 miR-148a expressing populations showed 60-80% reduction in the invasion potential as judged by the invasion of the cells through matrigel coated transwell inserts. On the other hand, the P1, P2 polyclonal population cells with exogenous NRP1 expression showed only marginal (~20%) reduction in the invasion potential upon doxycycline induced miR-148a expression (Figure 3A, B, D, G, H). Thus, restoration of NRP1 expression rescued the reduction in the invasion potential brought about by miR-148a expression, indicating the role of NRP1 in the invasion ability of medulloblastoma cells. Further, on subcutaneous injection, P2 polyclonal population of Daoy cells transfected with the NRP1 cDNA construct formed tumors of size comparable to that of the control vector cells after doxycycline induction of miR-148a expression (Figure 4A, D). Thus, NRP1 expression was found to rescue the reduction in tumorigenicity brought about by miR-148a expression indicating major role for NRP1 in miR-148a mediated tumorigenicity inhibition.

### NRP1 expression in medulloblastomas and its correlation with molecular subgroups and, survival

**Figure 6:**
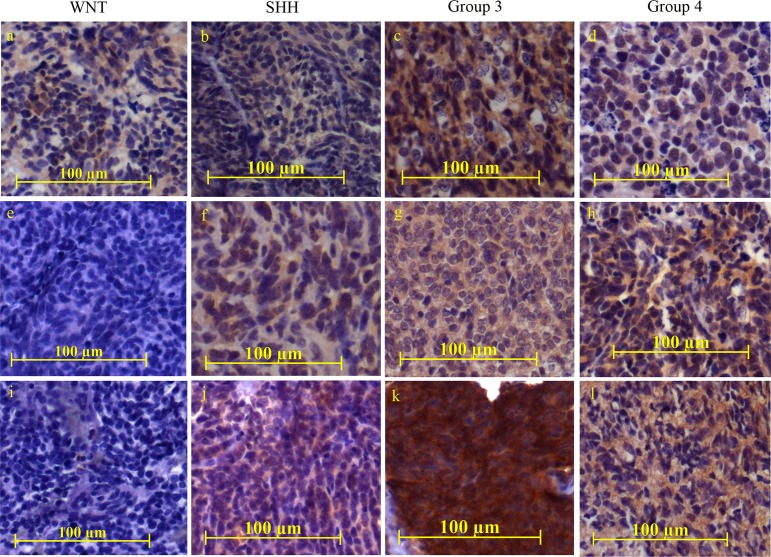
Immunohistochemical analysis of NRP1 expression in medulloblastoma tumor tissues NRP1 expression was studied in FFPE tissue sections belonging to the four molecular subgroups (WNT, SHH, Group 3, Group 4). The staining was scored as ‘negative’ for complete absence of staining, ‘Low’ for weak intensity and focal positive (~10-20% cells positive) areas, ‘Moderate’ for moderate intensity and more than or equal to 50% positive area while, ‘High’ for high intense staining in more than 80% of area. Representative images of NRP1 staining in the medulloblastoma tissues scored as negative (e, i), low (a, b, d, f, h), moderate (c, g, j, l) and high intense staining (k) respectively.

**Figure 7:**
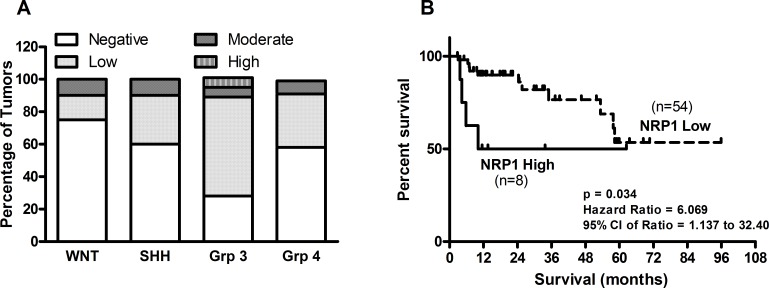
Expression of NRP1 across the four molecular subgroups of medulloblastomas (A) Percentage distribution of medulloblastoma tissues having NRP1 expressed scored as negative, low, moderate or high is shown across the four subgroups of medulloblastomas. (B) Kaplan Meier survival analysis of 62 medulloblastoma patients segregated into two groups based on NRP1 expression. “NRP1 low” group includes medulloblastoma tissues with no or low NRP1 expression while “NRP1 high” group includes tumor tissues with moderate or high NRP1 expression.

NRP1 expression was studied in a total of 93 medulloblastoma formalin fixed paraffin embedded (FFPE) tumor tissues by immunohistochemical analysis. Figure 6 shows representative photographs of NRP1 expression in medulloblastoma tissues belonging to the four molecular subgroups. Majority (75%) of the WNT subgroup medulloblastomas showed no detectable NRP1 expression, while only 23% Group 3 tumors lacked NRP1 expression (Figure 7A). Kaplan Meier survival analysis of 62 medulloblastoma cases, showed that tumors with moderate or high NRP1 expression had significantly poorer overall survival (p = 0.0349; hazard ratio 6.06) than those having no detectable or low NRP1 expression (Figure 7B).

## DISCUSSION

MiR-148a expression has been shown to be downregulated in various cancers including hepatocellular carcinoma, breast cancer, prostate cancer, pancreatic cancer, gastric cancer, non-small cell lung cancer, advanced colorectal cancer, adult acute lymphoblastic leukemia as well as in cancer associated fibroblasts in endometrial cancer [7, 8, 11]. Hypermethylation of CpG island in the miR-148a promoter region has been found to bring about downregulation of miR-148a expression in many cancers [7]. Expression of miR-148a at levels comparable to those in the WNT subgroup medulloblastomas or higher was found to inhibit growth and bring about reduction in the clonogenic potential, invasion potential and tumorigenicity of medulloblastoma cells belonging to the three subgroups *viz*. SHH, Group 3 and Group 4. MiR-148a expression has been reported to reduce growth, tumorigenic potential and invasion potential of various cancer cells including HBx expressing hepatocarcinoma cells, gastric cancer cells, pancreatic cancer cell lines and prostate cancer cell lines [11-14]. Recently miR-148a has also been reported to inhibit glioma stem cell-like properties and tumorigenicity [15, 16]. Thus, tumor suppressive effect of miR-148a expression in medulloblastoma cells is consistent with its role in other cancers.

Expression of ROCK1 and DNMT1, two known miR-148a targets, was found to be downregulated upon miR-148a expression in medulloblastoma cells [17, 18]. Rho-ROCK signaling cascade plays a major role in the re-organization of cytoskeleton during cell migration, adhesion, and cytokinesis [18]. Downregulation of DNMT1 expression has been shown to reduce tumor incidence in the *Ptch1* heterozygous knock-out mice, a mouse model of SHH subgroup medulloblastomas [19]. Downregulation of ROCK1 and DNMT1 upon miR-148a expression is therefore likely to contribute to miR-148a's tumor-suppressive effect on medulloblastoma cells.

Neuropilin 1 (NRP1) was identified as a novel target of miR-148a in the present study. 3′-UTR of the full length NRP1 transcript contains a single 8-mer miR-148a target site which is conserved among vertebrates (http://www.targetscan.org). Luciferase reporter assay validated NRP1 as a miR-148a target. Western blot analysis showed downregulation of full length NRP1 protein (120 kDa isoform) on miR-148a expression in multiple cell lines including Daoy, D283, medulloblastoma cell lines and U373 glioblastoma cell line, further confirming NRP1 as a miR-148a target. NRP1 gene encodes multiple alternately spliced transcripts. 5882 bp CCDS7177 transcript encodes 923 amino acid long full length protein whose 3′-UTR contains the miR-148a target site (http://ncbi.nlm.nih.gov/gene). Shorter CCDS31179, CCDS31180 transcripts encode 609 and 644 amino acid long proteins respectively, that lack the 3′-UTR as well as the transmembrane domain. These isoforms encode soluble NRP1 forms that have been shown to act as antagonists of the full length membrane bound NRP1 protein [20]. The 80 kDa NRP1 protein levels did not change upon miR-148a expression as judged by the western blot analysis. Therefore, it most likely belongs to the soluble NRP1 isoforms based on both its size and the real time RT-PCR analysis that shows the contribution of the shorter soluble isoforms to the total NRP1 expression levels. Thus, miR-148a targets only the functionally active full length NRP1 isoform and not the soluble isoforms that act as antagonist of NRP1 activity.

NRP1 is a single pass trans-membrane protein, which was first identified as a ligand binding co-receptor of Semaphorin 3A that plays a role in axon patterning in nervous system development [21, 22]. Subsequently, it was shown to act as a co-receptor of VEGF-A_165_ and other isoforms of VEGF that promote blood vessel growth during normal vasculature development and during angiogenesis in tumor progression [23]. NRP1 has also been reported to act as a co-receptor of multiple growth factors like TGF-beta 1, HGF, EGF and PDGF [24-28]. NRP1 has been reported to be over-expressed in various cancers including breast, prostate, pancreatic, colon, gliomas and its expression has been reported to correlate with poor prognosis [21, 29, 30]. In the present study, majority (75%) of the WNT subgroup medulloblastomas lacked detectable NRP1 expression. This observation is consistent with the high miR-148a expression in the WNT subgroup medulloblastomas as well as the low incidence of metastasis and excellent survival of the WNT subgroup patients [5, 6]. Majority of the Group 3 tumors which are known to have the worse survival among the four medulloblastoma subgroups expressed NRP1. Further, Kaplan-Meier survival analysis showed NRP1 expression in medulloblastomas to be associated with worse survival. Hence, NRP1 expression is a useful marker for prognostication in medulloblastomas.

Recently, Snuderl *et al* have reported that Placental Growth Factor (PIGF)-NRP1 signaling is necessary for growth and spread of medulloblastomas [31]. Inhibition of PIGF-NRP1 signaling was found to result in regression of medulloblastoma xenografts, decreased metastasis and increased survival of mice. NRP1 has been shown to promote epithelial mesenchymal transition and increase motility of endothelial cells as well as cancer cells by regulating TGF-beta, integrin and HGF/c-Met signaling [25, 32]. Downregulation of NRP1 expression has been shown to inhibit motility of medulloblastoma cells from *Ptch1* (+/−) mice [33]. Autocrine VEGF-VEGFR2-NRP1 signaling has also been reported to promote cancer stem cell maintenance in glioblastoma while NRP1 expression in breast cancer stem cells has been shown to be linked with the activation of NF-kB signaling [34, 35]. NRP1 has been shown to act as a co-receptor in multiple signaling pathways that promote tumor growth and progression [36]. Besides, the role of NRP1 in promoting tumor angiogenesis is very well established [37]. NRP1 has therefore been identified as an attractive therapeutic target for various cancers. In the present study as well, NRP1 expression was found to bring about almost complete rescue of miR-148a mediated inhibition of the invasion potential and tumorigenicity of medulloblastoma cells. This indicates major role for NRP1 in miR-148a's tumor-suppressive effect on medulloblastoma cells. Therefore, miR-148a mediated down-regulation of NRP1 could be an effective therapeutic strategy for the treatment of medulloblastomas as well as other cancers.

A number of approaches have been explored for targeting NRP1 including siRNAs against NRP1, anti-NRP1 antibodies, peptides targeting NRP1, and soluble NRP1 antagonists [38, 39]. NRP1 siRNAs or anti-NRP1 peptides have been shown to inhibit tumor growth, angiogenesis and metastasis in several cancers including hepatocellular carcinoma and glioblastoma [40-42]. Anti-NRP1 humanized monoclonal antibody MNRP1685A has been introduced in clinical trial for the treatment of various advanced solid tumors in combination with bevacizumab [43]. Circulating NRP1 is abundantly found in human plasma that is contributed by soluble NRP1 as well as the extracellular domain shed from the membrane bound full length isoform [44]. Therefore, any inhibitor that targets NRP1 protein would also target these soluble isoforms and hence would be required in large amounts for therapeutic effectiveness. However, since miR-148a targets only the NRP1 full length mRNA and not the soluble isoforms, it is likely to serve as a more effective strategy for targeting NRP1.

MiR-148a is expressed in normal tissues and its expression is known to be lost in several cancers often as a result of promoter hypermethylation [7]. MiR-148a expression at physiological levels showed tumor suppressive effect on medulloblastoma cells. Therefore, miR-148a as a therapeutic agent is unlikely to be toxic to normal tissues. MiR-148a was found to bring about tumor-suppressive effect by targeting NRP1, ROCK1 and DNMT1. These targets are distinct from those of other tumor-suppressive miRNAs like miR-34a and let-7a that are being explored for their therapeutic potential (www.mirnarx.com) [45]. MiR-148a is therefore an attractive therapeutic agent for the treatment of medulloblastomas as well as other cancers.

## MATERIALS AND METHODS

### Cell culture

Human medulloblastoma cell lines Daoy and D283 were obtained from ATCC, Manassas, VA, USA. Authenticity of the cell lines was confirmed by the Short Tandem Repeat (STR) marker profiling. Medulloblastoma cell line D425 was a kind gift from Dr. Darell Bigner (Duke University Medical Centre, Durham, NC, USA). The cells were grown in Dulbecco's Modified Eagle Medium: Nutrient Mixture F-12 (DMEM/F-12) supplemented with 10% Fetal Bovine Serum (FBS) (Life Technologies, Carlsbad, CA, USA) in a humidified atmosphere of 5% CO2. HEK293 FT cells were obtained from Life Technologies, Carlsbad, CA, USA and were grown in Dulbecco's Modified Eagle Medium (DMEM) supplemented with 10% FBS.

### Tumor tissues

Medulloblastoma tumor tissues were obtained with the approval of the Institutional Review Board. All the medulloblastoma cases studied were treated as per the standard practices with surgery followed by radiation (with the exception of less than 3 yr old children) and chemotherapy. The normal human cerebellar tissues were obtained from the Human Brain Tissue Repository at the National Institute of Mental Health and Neurosciences (NIMHANS), Bangalore, India.

### Transfection of medulloblastoma cells with synthetic miRNA mimics

D283 cells were transfected with 50 nM of miR-148a mimic, or siGLO (a RISC-free control siRNA) or siRNA negative control (Dharmacon, Thermo Scientific, Lafayette, CO, USA) as per the manufacturer's protocol for a period of 48 h using Dharmafect-2 transfection reagent. The transfected cells were seeded for various assays after 72 h. MiRNA levels in the transfected cells were estimated by real time Reverse Transcription-Polymerase Chain Reaction (RT-PCR) analysis using *RNU48* as an endogenous control.

### Transduction of medulloblastoma cells with miRNA expressing lentiviral vectors

Genomic region encoding miR-148a was amplified from normal human lymphocyte DNA by Polymerase Chain reaction (PCR) and cloned in a pTRIPZ lentiviral vector (Open Biosystems, Thermo Scientific, Lafayette, CO, USA) downstream of doxycycline-inducible minimal Cytomegalo virus (CMV) promoter. The nucleotide sequences of the primers used for amplification are given in Supplementary Table 1. Daoy, D425 and D283 medulloblastoma cell lines were transduced with pTRIPZ-miR-148a lentiviral particles and stable polyclonal populations were selected in the presence of puromycin. The cells transduced with lentiviral particles of empty pTRIPZ vector or pTRIPZ-NT vector expressing non-targeting shRNA (Open Biosystems, Thermo Scientific, Lafayette, CO, USA) were used as a control.

### Real time RT-PCR analysis and Molecular Classification of Medulloblastomas

MiR-148a expression in the transfected cells was determined by real time RT-PCR analysis using the Taqman assay (Applied Biosystems, Thermo Scientific, Lafayette, CO, USA) with *RNU48* as a control house-keeping small RNA. Relative quantification of the expression levels of *NRP1* was done by SYBR Green real time RT-PCR assay using *GAPDH* as a house-keeping control gene. Relative Quantity (RQ) was estimated as $RQ=2^{-(Ct}{{}_{test}}^{-Ct}{{}_{control}}^{)}\times 100$. The primers for real time PCR were designed such that they corresponded to two adjacent exons of the gene (Supplementary Table 1). In case the primers belonged to a single exon, the RNA was treated with RNase-free DNase before reverse transcription. Molecular classification of FFPE tumor tissues was carried out using real time RT-PCR as described before [6].

### MTT reduction assay

Growth of miRNA expressing cells and control cells was studied by MTT [2-(4,5-dimethyl-2-thiazolyl)-3,5- diphenyl-, bromide] reduction assay. 500 or 1500 cells of Daoy and D425 cell lines respectively, were seeded per well of a 96-well micro-titer plate [46]. Cell growth was followed over a period of 12 days with replenishment of the medium every 3^rd^ day. 20 μl of MTT (5 mg/ml) was added to each well at the end of the incubation period and the cells were incubated further for a period of 4 h. 100 μl of 10% SDS in 0.01 M HCl was added per well to dissolve the dark blue formazan crystals. Optical density was estimated using an Enzyme-linked immunosorbent assay (ELISA) reader at a wavelength of 540 nm with a reference wavelength of 690 nm.

### Clonogenic Assay for assessing clonogenic potential and radiation sensitivity

1000 cells were plated per 55 mm plate and were allowed to grow for 6-8 days until microscopically visible colonies formed. The cells were fixed by incubation in chilled methanol:acetic acid mixture (3:1) and the colonies were visualized by staining with 0.5% crystal violet. For radiation sensitivity determination, the cells were irradiated at a dose of 4 Gy (Cobalt-60 gamma irradiator, developed by Bhabha Atomic Research Centre, India) 16 h after seeding for the clonogenic assay.

### Soft agar colony formation assay

7500 Daoy cells or 1000 D425 cells were seeded in DMEM /F-12 medium supplemented with 10% FBS containing 0.3% agarose over a basal layer of 1% agarose in DMEM/F-12/10%FBS. The cells were incubated for about 3-4 weeks and the colonies formed were counted.

### *In vitro* invasion assay

50,000-75000 cells were seeded in 200 μl of plain DMEM in the upper chamber of 8-μm pore size transwell inserts (BD Biosciences, San Hose, CA, USA) coated with Matrigel^TM^, placed in a 24 well micro-titer plate. 750 μl of DMEM supplemented with 10% FBS was added to the lower chamber. The cells were allowed to migrate for 36-56 h and were labeled with Calcein AM (Life technologies, Carlsbad, CA, USA), a fluorescent dye, 1h prior to terminating the invasion. Non-invaded cells from the upper chamber were removed by wiping the upper portion of the insert with a cotton bud. The inserts were photographed using a Zeiss Axiovert 200M fluorescence microscope. Fluorescence intensity of the Calcein AM-labeled cells on the lower side of the insert was measured using a fluorescence reader using excitation wavelength of 488 nm and emission wavelength of 525 nm.

### *In vivo* tumorigenicity assay using subcutaneous xenografts in immunodeficient mice

The experimental protocol was approved by the Institutional Animal ethics committee. 5 × 10^6^ Daoy/D425 cells transduced with pTRIPZ-miR-148a construct were injected subcutaneously in the flanks of BALB/c Nude mice (CAnN.Cg-*Foxn1nu*/Crl strain received from Charles River, USA) following doxycycline induction for 72 h. Control cells transduced with pTRIPZ-NT construct were injected into the other flanks of the mice following doxycycline induction. Size of the tumors developed was measured using Vernier caliper at regular intervals over a period of 1-2 months.

### *In vivo* tumorigenicity assay using orthotopic xenografts in immunodeficient mice

D283 polyclonal populations transduced with pTRIPZ-miR-148a construct were transfected with a pcDNA3.1 vector expressing firefly luciferase under CAG (CMV early enhancer/chicken beta-actin promoter) promoter. 2 × 10^5^ cells were injected into the cerebellum of BALB/c Nude mice through 0.5 mm burr hole in the midline, 2 mm posterior to lambda at 2 mm depth, using small animal stereotaxic frame (Harvard Apparatus, Holliston, MA, USA). The tumor growth was monitored by *in vivo* bioluminescence imaging. At the time of imaging mice were anesthetized using Ketamine (90 mg/kg body weight) and, Xylazine (20 mg/kg body weight). Serial images of the mice were captured after intraperitoneal injection of D-Luciferin (150 mg/kg body weight) using IVIS Spectrum (Perkin-Elmer, Waltham, MA) *in vivo* imaging system until the peak luciferase activity was attained. The photon output from the image with peak luciferase activity was quantified by manually drawing region of interest (ROI) around the luminescent source using “Living Image” software and expressed in radiance (photons/sec/cm^2^/steridian). A pseudo-color image representing light intensity (blue least intense and red most intense) was generated and superimposed over the gray scale reference image. All the animals were imaged on first and fourth week post intracranial injection.

### Luciferase reporter Assay

3′-UTR (untranslated region) of each of the potential miRNA target genes was amplified from genomic DNA of normal human peripheral blood lymphocytes using Phusion Taq polymerase (Thermo scientific, Pittsburgh, PA, USA). The 3′-UTRs were then cloned downstream of firefly luciferase cDNA from PGL3 vector (Promega, Madison, WI, USA) in a pcDNA 4.0 plasmid vector (Invitrogen, Life Sciences, Carlsbad, CA, USA). The genomic region encoding miR-148a was cloned into pcDNA 3.0 plasmid vector wherein the miRNA was expressed under CMV promoter in mammalian cells. HEK 293T cells were transfected with the luciferase reporter plasmid, miRNA expressing plasmid and a plasmid vector expressing EGFP fluorescent protein by Calcium phosphate BES buffer method. Luciferase activity was assessed from the total protein extracted from the transfected HEK293T cells and was normalized against the EGFP fluorescence measured using Mithras LB940 multimode reader (Berthold Technologies, Bad Wildbad, Germany).

### Western blotting

Total protein extracts of the medulloblastoma cell lines before and after expression of miR-148a, were separated by SDS-PAGE electrophoresis and blotted onto a PVDF membrane (Amersham-GE Healthcare Life Sciences, Mumbai, India). The membrane was then incubated with anti-NRP1 (#3725), anti-DNMT1 (#5119), anti-ROCK1 (#4035) antibody (Cell signaling technologies, Boston, MA, USA) or anti-γ-tubulin (T3559) antibody (Sigma-Aldrich, MO, USA) as per the manufacturer's instructions, followed by incubation with anti-rabbit IgG conjugated with Horse Radish Peroxidase and developed using SuperSignal West Pico chemiluminescent substrate (Pierce, Thermo Fischer scientific, Waltham, MA, USA). Protein expression levels were determined by intensity measurements of the bands corresponding to the proteins of interest using ImageJ software (http://imajeJ.nih.gov.in).

### Immunohistochemical Analysis

5 μm sections of FFPE medulloblastoma tissues were deparaffinized, rehydrated and the antigen retrieval was done as per the protocol (http://www.cellsignal.com). Non-specific binding to the sections was blocked using 3% Bovine serum albumin (BSA) for 1 h. The sections were incubated in 1:100 diluted anti-NRP1 antibody (Cell signaling technologies, Boston, MA, USA) in 1% BSA overnight at 4ºC, followed by incubation with the peroxidase conjugated secondary anti-rabbit IgG (Pierce, Thermo Fischer scientific, Waltham, MA, USA). As a negative control, the sections were treated identically but without the primary antibody. The bound peroxidase signal was detected using 3, 3-' Diaminobenzidine as a substrate. The staining was visualized and photographed using Zeiss Upright fluorescent microscope Axioimager. Z1 and scored on the basis of the intensity and percentage of positive cells. The scoring of the staining intensity by a neuropathologist blinded to the molecular subgroup or survival data.

### Correlation of miR-148a and NRP1 expression with molecular sub-grouping and Survival

MiR-148a levels determined by real time RT-PCR analysis as described before were correlated with molecular sub-grouping of 141 FFPE medulloblastoma tumour tissues studied. The significance of the difference in miR-148a expression in the WNT subgroup tumors as compared to other medulloblastomas was evaluated by the Student's t test. NRP1 expression evaluated by immunohistochemical analysis was correlated with molecular sub-grouping. Kaplan-Meier survival analysis was done using GraphPad Prism version 5.01 software and the significance of the difference in the NRP1 high and NRP1 low groups was determined by Log-Rank test.

All the above experiments were performed at least in triplicate. Student's t test was performed to check for statistical significance of the difference in the performance of miR-148a expressing cells as compared to the control cells.

## SUPPLEMENTARY MATERIALS FIGURES AND TABLE


