# Supplementary material for: MiR-148a, a microRNA upregulated in the WNT subgroup tumors, inhibits invasion and tumorigenic potential of medulloblastoma cells by targeting Neuropilin 1
Source: Oncoscience. 2015 Mar 2;2(4):334–48. doi: 10.18632/oncoscience.137 (PMC4468320; doi:10.18632/oncoscience.137)
Supplement: Supplementary file 1 [file oncoscience-02-0334-s001.pdf]

MiR-148a, a microRNA upregulated in the WNT subgroup tumors, inhibits invasion and tumorigenic potential of medulloblastoma cells by targeting Neuropilin 1

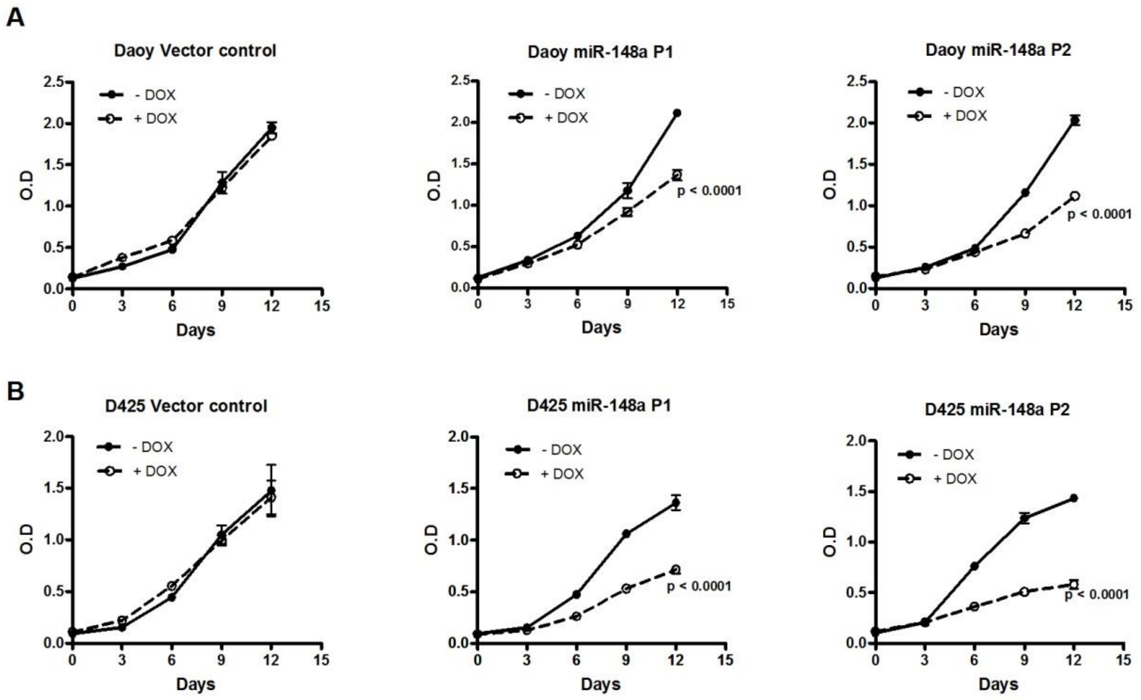

Supplementary Figure S1: Effect of miR-148a expression on growth of medulloblastoma cell lines Daoy (A) and D425 (B) studied using MTT assay. The figure shows representative experiments using P1 and P2 polyclonal populations stably transduced with pTRIPZ-miR-148a construct and the control vector construct.

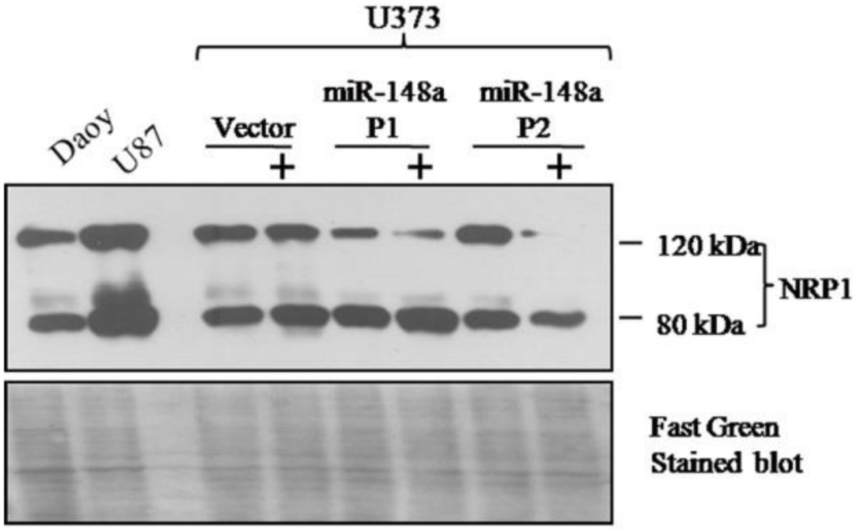

Supplementary Figure S2: Western blot analysis showing NRP1 protein levels in U373 glioblastoma cell line stable polyclonal populations transduced with control pTRIPZ vector and pTRIPZ-miR-148a construct.

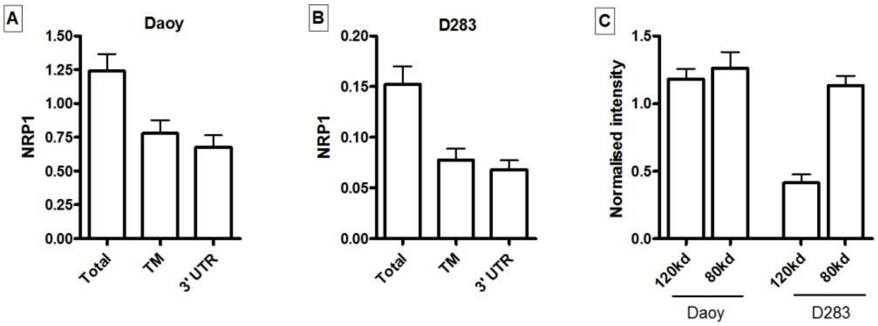

Supplementary Figure S3: The relative levels of NRP1 mRNA and protein levels in Daoy and D283 cells. NRP1 mRNA levels were estimated by real time RT-PCR using NRP1 specific primers to amplify all known transcripts (total), primers specific for Transmembrane domain (TM) and primers specific for the 3'-UTR region of the full length NRP1 transcript in Daoy (A) and D283 (B) cells. (C). Relative proportion of NRP1 protein levels corresponding to full length 120 kDa and shorter isoform 80 kDa in Daoy and D283 cells as estimated by image analysis of the western blots separating total protein extracts of the cell lines and probed with anti-NRP1 antibody.

Supplementary Table 1. The nucleotide sequences of the primers used for real time RT-PCR analysis and cloning of genomic regions of miR-148a, and 3'-UTR of the indicated miR-148a target genes.

Primers used in the study –

miR-148a genomic region cloning	FWD	AAGGCTGCAGAGTGTGCGATTCTTGCAG
	REV	AATTCTGCAGAAATTCTACAGTCAGGAGTC
NRP1 (all isoforms)	FWD	CCGCCTGAACTACCCTGAGAA
	REV	GCCCAAGTCTACCTGTATCCAC
NRP1-transmembrane	FWD	CCTCATCACCATCATAGCCA
	REV	AGCACGACCCCACAGACAGC
NRP1-3'UTR	FWD	ACAAGGGAAGTGGAAGGAAGGAA
	REV	AAAATACTTGACCCCCAGGC
NRP1 3'UTR cloning	FWD	CCTGAATTCGTACTTGTGTATAATTGCCCGC
	REV	TTATCTAGATGTTTAATTCAACTGTTTCTTTGGA
NRP1 3'UTR SDM	FWD	CAACTTTAAAATTTAAAGTATCTTGTCTAGATAAATATATTTAAAAATATATG
	REV	CATATAATTTTTAAATATATTTATCTAGACAAGATACTTTAAATTTTAAGTTG
DNMT1 3'UTR cloning	FWD	GACGAATTCTGCCCTCCCGTCACCC
	REV	TAATCTAGACTCATACAGTGGTAGATTTG
ROCK1 3'UTR cloning	FWD	GCGGGATCCCCTACAGGTAGATTAGATTA
	REV	CTCTCTAGATGAGCACAGAGTCATTAGT
ARHGAP21 3'UTR cloning	FWD	ACTGGGGGTATGTCCACTCTA
	REV	ATTTCCAGTGTTTAATTGGGTATGC
TMSB10 3'UTR cloning	FWD	CAGGAATTCAGCAGGAGAAGCGGAGTGAAA
	REV	CCACTCGAGCCATGCCACGAGGTGTGT
